# intmap: fast and flexible mapping of mobile DNA integration for basic and translational research

**DOI:** 10.1093/bioinformatics/btag310

**Published:** 2026-05-18

**Authors:** Gregory J Bedwell, Peter Cherepanov, Alan N Engelman

**Affiliations:** Department of Cancer Immunology and Virology, Dana-Farber Cancer Institute, Boston, MA 02215, United States; Department of Medicine, Harvard Medical School, Boston, MA 02115, United States; Chromatin Structure and Mobile DNA Laboratory, The Francis Crick Institute, London, NW1 1AT, United Kingdom; Department of Cancer Immunology and Virology, Dana-Farber Cancer Institute, Boston, MA 02215, United States; Department of Medicine, Harvard Medical School, Boston, MA 02115, United States

## Abstract

**Summary:**

Integration of exogenous DNA into host cell chromatin is a hallmark of many virus and transposon lifecycles and the foundational basis for many modern cellular and genetic therapies. Defining integration sites (ISs) in a population of cells can accordingly inform fundamental aspects of mobile DNA biology and therapeutic treatment outcomes. Here, we describe intmap, a software that generalizes IS analysis to diverse data-types and experimental systems. intmap functions independently of strict library design assumptions and is highly tunable with respect to analysis parameters. Using both simulated and experimental data, we show that IS mapping with intmap is fast, accurate, and highly flexible.

**Availability and implementation:**

intmap, related documentation, and a convenient installation script are available at https://github.com/gbedwell/intmap. Additional information is provided in the online Supplement. Analysis commands, outputs, and other information related to this manuscript are available at https://doi.org/10.5281/zenodo.19475929.

## Introduction

Mobile DNA elements such as retroviruses and retrotransposons have significantly impacted Metazoan evolution (Craig *et al.* 2002). In these systems, integration can occur in numerous genomic locations, with multifactorial host-element interactions guiding the selection of particular target DNA acceptor sites (Kvaratskhelia *et al.* 2014, Sultana *et al.* 2017, Bourque *et al.* 2018, Jóźwik *et al.* 2022, Sabatino *et al.* 2022, Singh *et al.* 2022). Certain DNA viruses, such as adenoassociated virus (AAV), can additionally leverage double strand DNA breaks for integration (Miller *et al.* 2005, Hanlon *et al.* 2019, Breton *et al.* 2020, Nguyen *et al.* 2021, Van Gorder *et al.* 2023). The locations of integration events can profoundly impact cell biology, viral disease progression/persistence, cancer risk, therapeutic efficacy, and other medically-related outcomes (Howe *et al.* 2008, Johnson 2019, Nobles *et al.* 2020, Bedwell *et al.* 2021, Biasco *et al.* 2021, Coffin *et al.* 2021, Lian *et al.* 2021, Senft and Macfarlan 2021). As such, precise mapping of integration events is of interest in both basic and translational research.

**Figure 1:**
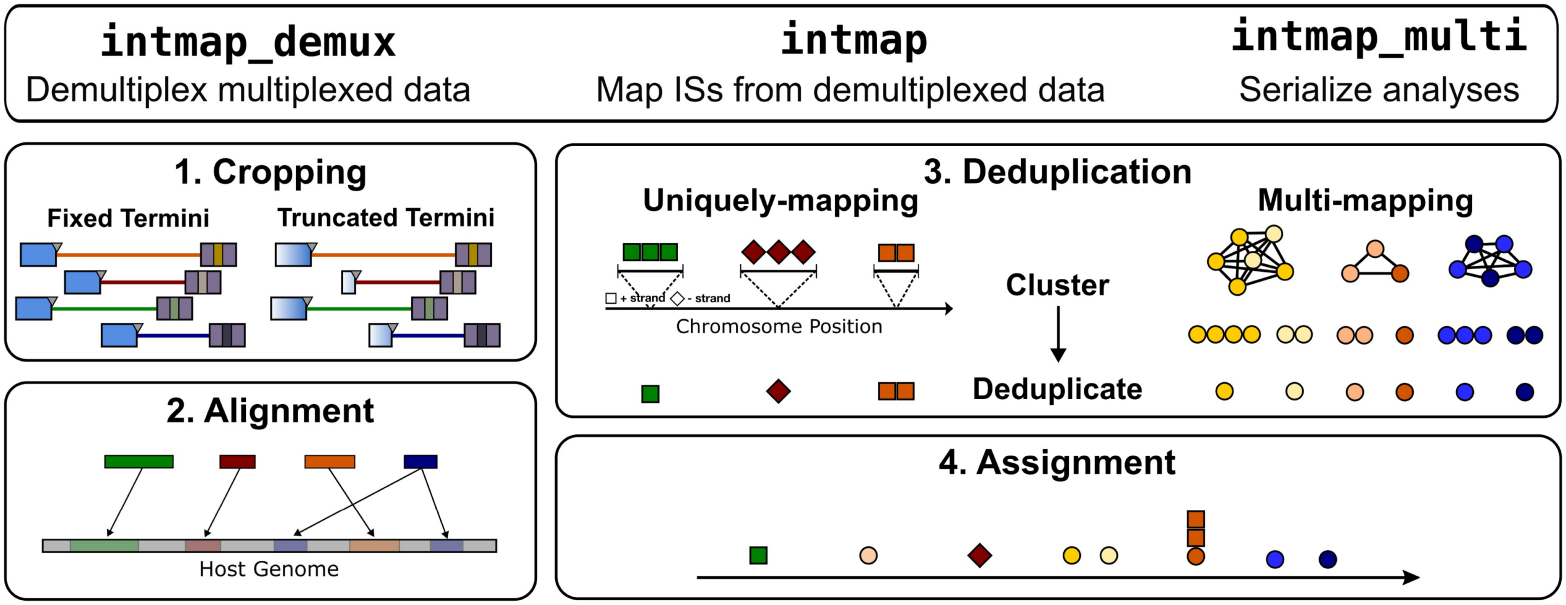
Schematic overview of IS mapping with intmap. The different intmap modules are listed in the top section. The four primary mapping steps—(i) cropping, (ii) alignment, (iii) deduplication, and (iv) assignment—are enumerated in the remaining sections. Cropping involves the removal of integrant and linker sequences (medium-blue and purple-gray boxes, respectively) from the sequenced fragments. Distinct UMIs are denoted as multi-colored bars within linkers. For integrant ends of variable length (referred to as truncated termini), the position along the integrant end is indicated according to the depth of color, with progression into darker colors indicating more full-length termini. Host genome fragments are represented as colored horizontal lines between integrant and linker sequences. The position of the integrant-host junctions (i.e. the genomic position that is ultimately mapped) are indicated by gray arrowheads. Alignment involves aligning cropped reads to the target genome. By default, intmap retains both uniquely-mapping (dark green, dark red, and dark orange bars) and multi-mapping (dark blue bar) fragments. Aligned fragments undergo quality control checks and are designated as either uniquely- or multi-mapping during these checks. For deduplication—the process of removing duplicates from the aligned fragments—similar fragments are clustered together according to either their aligned coordinates (uniquely-mapping fragments) or sequence similarity (multi-mapping fragments). Multi-mapping fragment clustering proceeds in multiple steps to sequentially reduce the search space for sequence similarity calculations. When UMIs are present, fragment clusters are sub-clustered based on UMI identity. A representative fragment from each (sub-)cluster is then chosen. In the deduplication diagram, uniquely-mapping fragments are represented as colored squares (plus strand fragments) or diamonds (minus strand fragments). Similar uniquely-mapping fragments are depicted in clusters originating from nearby genomic positions (brackets; uniquely-mapping fragment colors match Step 2 colors). Multi-mapping fragments are diagrammed as colored circles. For illustrative purposes, the multi-step clustering routine for multi-mapping fragments expands beyond the single multi-mapping fragment from Step 2 (dark blue bar/circles). Finally, retained fragments are assigned to their final genomic positions. Assignment criteria are further described in the text. Multi-mapping fragment reassignment is in part depicted as dark orange squares and circles being assigned to the same genomic position. Following mapping, auxiliary outputs, such as IS overlap with defined genomic features and peak-calling for Cas-mediated AAV integration hotspots are optionally available.

Modern integration site (IS) mapping approaches utilize massively parallel sequencing platforms to simultaneously map thousands of events in a single experiment (Serrao *et al.* 2016, Afzal *et al.* 2017, Sherman *et al.* 2017, Spinozzi *et al.* 2017, Afzal *et al.* 2020, Breton *et al.* 2020, Wells *et al.* 2020, Nguyen *et al.* 2021, Yan *et al.* 2023). IS libraries are most commonly prepared using ligation-mediated (LM) polymerase chain reaction (PCR), the closely related linear amplification-mediated (LAM)-PCR, or integration site loop amplification [ISLA; see Wells *et al.* (2020), Gabriel *et al.* (2014), and Wagner *et al.* (2014), respectively]. The final products of LM- and LAM-PCR are essentially identical: linear DNA molecules containing a terminal portion of the mobile element (i.e. the integrant), a portion of the host genome directly adjoining the integrant, and a specific linker sequence ligated to the opposing end of the genomic fragment (see Fig. 1, Step 1). Notably, the final library products for other sequencing approaches aimed at identifying chromosomal translocation hotspots (e.g., LAM-HTGTS) and sites of CRISPR/Cas off-target cleavages (e.g., GUIDE-seq, DISCOVER-seq, and CIRCLE-seq) share this same general structure (Tsai *et al.* 2015, 2017, Hu *et al.* 2016, Wienert *et al.* 2020). ISLA similarly produces linear DNA fragments that harbor the integrant terminus on one end. Unlike with LM- and LAM-PCR-based approaches, however, the non-integrant end harbors the reverse compliment of a portion of the integrant terminus itself (Wagner *et al.* 2014). Other differences between IS library protocols can arise with respect to features such as the presence of unique molecular identifiers (UMIs), UMI structure and position, and the designed sequencing orientation of fragments [compare, e.g., the library designs described in Serrao *et al.* (2016), Wells *et al.* (2020), Yan *et al.* (2023), and Sherman *et al.* (2017)]. The utilization of different library architectures has led to the development of distinct analysis pipelines compatible with particular library designs but incompatible with others. These pipelines additionally differ in other key ways, such as the genome builds that they support, the data-types that they can analyze, the integrants that they can reliably map, and the manner in which they handle fragments that align equally well to multiple genomic positions (hereafter referred to as multi-mapping fragments).

Here, we describe intmap, a collection of Python-based command-line interface (CLI) modules intended to generalize IS analysis. intmap differs from existing IS mapping software in several important ways. First, intmap supports parallelization of resource-intensive tasks across Unix-based systems. Second, intmap is highly flexible with respect to the data-types and library designs that it accepts. Third, intmap can accurately map IS locations of diverse integrant species, including retroviruses and AAV, which differ markedly in the nature of their terminal sequences. Fourth, intmap enables the tunability of a large number of mapping parameters to meet diverse analytical needs. Fifth, intmap efficiently and scalably processes and retains both uniquely- and multi-mapping fragments and assigns multi-mapping fragments to probable genomic locations without requiring manual inspection. Finally, intmap incorporates both new and previously described methods to minimize experimental artifacts.

## Implementation

intmap’s functionality is implemented across three separate CLI modules: intmap_demux for demultiplexing multiplexed data, intmap for mapping ISs to genomic positions, and intmap_multi for automated serialization of multiple analyses (Fig. 1). External software requirements are listed in Section 1.1 of the Supplementary Material.

## Demultiplexing

intmap_demux can demultiplex multiplexed data based on sample-specific in-line indexes (barcodes), header-based indexes, or separate index files. See Section 1.2 of the Supplementary Material for additional information.

## Mapping

Mapping of demultiplexed reads to genomic locations proceeds in four key steps: cropping, alignment, deduplication, and assignment (Fig. 1). Each of these are individually described in the sections below.

## Cropping

Techniques used for IS library generation rely on known sequences on both termini of DNA fragments for PCR amplification. These sequences must be identified and removed from each read in order to accurately define integrant-host junctions (Fig. 1, Step 1). intmap’s cropping routine identifies user-defined end sequences (e.g., integrant and linker sequences) in each read in the input file(s). When these sequences are found, the sequence of the given read up to and including the match positions are removed and the remaining sequence is saved to a new FASTQ file. UMIs are extracted during cropping. When UMIs are absent, they are defined as “N” for use in downstream processing. Additional information is given in Section 1.3 of the Supplementary Material.

## Alignment

Alignment proceeds in three stages. Reads are first aligned to a user-defined integrating-element sequence (retrovirus, transposon, etc.) to identify and remove reads arising from internal priming, plasmid contamination, or auto-integration. Reads that align in this step are discarded. Reads that pass step 1 are aligned to a user-defined reference genome (Fig. 1, Step 2). For short-reads, Bowtie2’s end-to-end algorithm is used for alignment (Langmead and Salzberg 2012), while long-read alignment utilizes minimap2 (Li 2018). Finally, aligned reads are processed for quality. The user is able to define the minimum- and maximum-allowed fragment lengths, the maximum mismatch rate, the maximum indel rate, the minimum mapping quality (MAPQ) score, the minimum average Phred quality score, and the maximum number of bases on the integrant-end before which there is an exact match to the reference genome. In intmap’s quality control (QC) routine, soft-clipped and ambiguous bases are treated as mismatches. Only aligned reads that pass the QC checks are carried on for further processing. Aligned fragments are stratified as uniquely- or multi-mapping during QC (Fig. 1).

## Deduplication

Deduplication procedures differ for uniquely- and multi-mapping fragments. For the former, fragments with identical alignment positions and UMIs are first grouped together and the fragment with the highest MAPQ and average Phred scores is chosen as the representative fragment. Next, “fuzzy” deduplication groups together uniquely-mapping fragments whose start and end coordinates fall within a user-defined window (Fig. 1, Step 3). A similar approach is used in Wells *et al.* (2020). The UMIs of grouped fragments are then compared using a directional clustering algorithm similar to the “directional” algorithm in UMI-tools (Smith *et al.* 2017). More information on UMI deduplication is given in Section 1.4.1 of the Supplementary Material. For multi-mapping fragments, the aligned fragment position is uninformative; the sequences themselves are the only reliable source of information. Multi-mapping fragment deduplication begins by collapsing fragments with the same sequences and UMIs into a single representative sequence. For paired-end reads, concatenated read-pairs are used for sequence comparisons. Next, representative fragments are compared for sequence similarity (Fig. 1, Step 3). intmap attempts to minimize the search space for each sequence by clustering similar sequences together prior to quantifying exact sequence similarity (Fig. 1, Step 3; see Section 1.4.2 of the Supplementary Material for more information). Ultimately, sufficiently similar fragments are grouped together as fuzzy matches, and UMI deduplication proceeds as for uniquely-mapping fragments. Only deduplicated fragments are carried on for further processing (Fig. 1, Step 3). These deduplication procedures work similarly on data with and without UMIs, providing a consistent deduplication workflow for IS analysis independent of library design.

## Assignment

Following deduplication, fragments are assigned to their final genomic locations (Fig. 1, Step 4). Uniquely-mapping fragments are assigned to their aligner-designated positions. For multi-mapping fragments, intmap can assign final positions in two distinct ways. The first way, which works similarly to the approach for uniquely-mapping fragments, is intended for situations where multiple copies of the same insertion (e.g., in clonally expanded cell populations or site-specific integration) are unexpected. The aligners used in intmap randomly choose one of the possible best-scoring alignment positions as the primary multi-mapping fragment position. While the ISs derived from these randomly chosen positions are not unambiguously defined, their retention works to accurately reflect the degree of integration stemming from multi-mapping fragments in annotated genomic regions (see Section 2.1.1 of the Supplementary Material). With 53.9% of the human genome classified as repetitive in the telomere-to-telomere assembly, the ability to quantify integration within repetitive regions is critical to obtain comprehensive views of integration across different contexts/systems (Hoyt *et al.* 2022). This approach to multi-mapping fragment position assignment, however, may under-represent any clonal or site-specific IS populations that are partially or completely composed of multi-mapping fragments. To address this limitation, intmap can alternatively assign multi-mapping fragment positions based on sequence similarity with uniquely-mapping fragments or other multi-mapping fragments with distinct UMIs and/or linker ligation positions (“multi-mapping fragment reassignment”; see Section 1.5.1 of the Supplementary Material for more information). This assignment mechanism, suitable when clonal expansion or site-specific integration are expected, helps to accurately represent positionally-biased IS populations in the output (see Section 2.1.2 of the Supplementary Material). When all fragments have been processed, intmap makes a final pass through the assigned fragment positions to tidy the final data, which is described in more detail in Section 1.5.2 of the Supplementary Material.

## Multiple analyses

intmap_multi serializes multiple analyses in a single command. It is written to be highly modular, allowing different analysis parameters to be defined within the same set of serialized analyses. intmap’s GitHub repository contains more information on the structure of the input file used by intmap_multi. Additional examples are provided on Zenodo.

## Results

intmap accurately mapped ISs across diverse data-types, library designs, and integrant-types (Figures S1C-E, S2B-D, S3B-C, S4A-B, and S5A-C). These data-types included paired-end short-read, single-end short-read, and long-read data (Figures S1C-E, S2B-D, S3B-C, S4A-B, and S5A-C). The integrant-types included fixed-end integrants (e.g., retroviruses; Figures S1C-E, S2B-D, S3B-C, and S4A-B) and randomly truncated integrants (e.g., AAV; Figures S5A-C). Parallelization of analyses across multiple cores decreased runtime several-fold (Figure S1A). intmap was moreover ∼6-fold faster than the state-of-the-art IS mapping software INSPIIRED, while maintaining mapping accuracy (Figure S3A-C). Finally, intmap efficiently and robustly handled multi-mapping fragments, mapping multi-mapping fragments without substantially impacting runtime and correctly identifying clonal populations comprised entirely of multi-mapping fragments (Figures S1A-D and S2A-D).

## Summary

Altogether, intmap generalizes IS analyses. intmap’s agnostic approach to library design specifications works to democratize IS analysis, freeing users from rigidly defined read structures and providing a consistent framework for robust IS analysis across systems. It moreover enhances the possibility of cross-study comparisons and meta-analyses of IS data. The tunability of intmap’s analysis parameters additionally provides analytical flexibility to better meet varying analytical needs. Finally, intmap’s applicability to diverse integrant-types establishes it as a proverbial Swiss Army knife for IS analysis. We expect that intmap will become a valuable tool for research across fields related to genomic editing and genomic integration.

## Supplementary Material

btag310_Supplementary_Data

## Data Availability

intmap is available at https://github.com/gbedwell/intmap. Small example datasets, along with analysis commands, and enumeration of all accepted arguments, are provided on GitHub. Commands and output for other analyses described here, along with related information, are available at https://doi.org/10.5281/zenodo.19475929.
