# Supplementary material for: intmap: fast and flexible mapping of mobile DNA integration for basic and translational research
Source: Bioinformatics. 2026 May 18;42(6):btag310. doi: 10.1093/bioinformatics/btag310 (PMC13275135; doi:10.1093/bioinformatics/btag310)
Supplement: btag310_Supplementary_Data [file btag310_supplementary_data.pdf]

Supplemental Material for ‘intmap: fast and flexible mapping of mobile DNA integration for basic and translational research’

Gregory J. Bedwell Peter Cherepanov Alan N. Engelman

1 Additional implementation information

1.1 Requirements

intmap was built using Python 3.11 and relies on several external packages and other stand-alone software, including: biopython v1.84 (Cock et al. 2009), joblib v1.4.2 (The joblib Developers (2024)), regex v2024.5.15 (Barnett (2024); https://github.com/mrabarnett/mrab-regex), RapidFuzz v3.10.1 (Bachmann (2024); https://github.com/rapidfuzz/RapidFuzz), numpy v2.1.2 (Harris et al. 2020), faiss-cpu v1.9.0 (Yamaguchi (2024); https://github.com/faiss-wheels/faiss-wheels), datasketch v1.6.5 (Zhu et al. (2024)), pybloom-live v4.0.0 (Fox (2024); https://github.com/joseph-fox/python-bloomfilter), pandas v2.2.3 (McKinney 2010; The Pandas Development Team 2024), statsmodels v0.14.4 (Seabold and Perktold 2010), scipy v1.15.2 (Virtanen et al. 2020), pyranges v0.1.4 (Mariotti, n.d.), ruptures v1.1.10 (Truong, Oudre, and Vayatis 2020), mmh3 v5.2.1 (Senuma 2025), parasail v1.3.4 (Daily 2016), samtools v1.21 (Li et al. 2009; Danecek et al. 2021), bowtie2 v2.5.4 (Langmead and Salzberg 2012), cutadapt v4.9 (Martin 2011), pysam v0.22.1 (The pysam Development Team (2024); https://github.com/pysam-developers/pysam), bedtools v2.31.1 (Quinlan and Hall 2010), seqtk v1.5-r133 (Heng Li (2025); https://github.com/lh3/seqtk), and minimap2 v2.29 (Li 2018). An installation script is provided in the GitHub repository that creates an intmap conda environment and installs all dependencies.

1.2 Demultiplexing

intmap_demux can demultiplex reads using either in-line indexes (barcodes), header indexes, or indexes stored in separate index FASTQ files. For all indexes, mismatches are allowed up to a user-defined error rate. Insertions and deletions (indels) are not allowed. For short-read paired-end data, intmap_demux can find in-line indexes on the 5′ ends of read 1 and read 2 together, read 1 only, or read 2 only. For long-read data, indexes can be found on the 5′ or 3′ ends of reads. Single-end short-read data should only contain in-line indexes on the 5′ ends of reads. In all cases, the software expects the indexes to be anchored (i.e., at the start/end of reads). If indexes in a given experiment are not anchored, an appropriate number of N’s can be appended to the provided index sequence and given to the software (i.e., on the left- and right-hand side of 5′ and 3′ indexes, respectively) to effectively anchor them while allowing ambiguity in the terminal non-index positions.

Lastly, intmap_demux does not remove any sequence from index-containing reads, as index portions of reads are automatically removed during the cropping step of IS mapping.

1.3 Cropping

Integrant- and linker-end sequences are both provided to intmap by the user. Each sequence should be at least 10 nucleotides. intmap utilizes cutadapt to identify and remove the defined integrant and linker sequences in respective reads. Reads that do not contain the expected sequence(s) are discarded. Reads that do contain the expected sequence(s) are subsequently examined for the reverse compliment of the other user-defined sequence to account for read-through of short fragments. To find integrants whose termini may have been randomly truncated during integration (as is common during AAV integration), the software looks for progressively shorter versions of the full-length user-defined search sequence. Shortening proceeds from the $3'$ end of the search sequence. The $5'$ end is assumed to be anchored at some common position (e.g., the primer binding site). All identified integrant and linker sequences are appended to the headers of the cropped FASTQ files with the OX tag for use in downstream processing. When appended, the respective integrant and linker sequences are separated by a hyphen.

When present, intmap extracts unique molecular identifiers (UMIs) from sequenced reads during cropping. UMI positions are defined by three parameters: presence on the integrant- and/or linker-end of the fragment, the expected UMI position relative to the provided integrant- or linker-end search sequence (expressed as the number of nucleotides between the integrant/linker sequence and the UMI), and UMI length. Extracted UMIs are appended to the headers of the cropped FASTQ files with the RX tag. When reported, integrant- and linker-end UMIs are separated by a hyphen. When integrant- and/or linker-end UMIs are absent, their values are defined as "N" for use in downstream processing.

1.4 Deduplication

1.4.1 UMI deduplication

Directional UMI comparisons in intmap begin by concatenating integrant- and linker-end UMIs into a single composite UMI. For each fragment cluster, reads with identical UMIs are grouped together. A graph of UMI neighbors is then constructed by enumerating all single-position substitution variants for each unique UMI and checking for their presence in the observed UMI set. This efficiently identifies all UMI pairs with a Hamming distance of 1, in accordance with the first condition of UMI-tools' directional algorithm (Hamming 1950; Smith, Heger, and Sudbery 2017). Next, directed edges are added between UMI neighbors that satisfy the count-based directionality condition of the directional algorithm ($c_a \geq fc_b - 1$, where c_a and c_b are the counts of the higher- and lower-abundance UMIs, respectively, with $f = 2$ by default; Smith, Heger, and Sudbery (2017)). Connected components are then extracted from the resulting adjacency graph. Within each extracted group, a directed breadth-first search (BFS) is performed from the highest-count UMI, with all UMIs connected by transversable edges being grouped into a single representative UMI (Cormen 2009). The BFS is repeated starting from the next-highest-count unassigned UMI until all UMIs have been assigned to a representative group.

1.4.2 Multi-mapping read deduplication

Multi-mapping read deduplication relies on sequence similarity. To reduce the computational burden of all-against-all sequence comparisons, intmap first generates MinHash fingerprints for each multi-mapping sequence (Broder et al. 2000). These fingerprints are subsequently converted into byte-representations for use with Faiss's binary index search, which is used to efficiently calculate the Hamming distances between fingerprints (Douze et al. 2024; Johnson, Douze, and Jégou 2017). The calculated Hamming distances are then used for fast estimation of the Jaccard index between fingerprints ($J \approx 1 - \frac{2d_H}{L}$, where J is the Jaccard index, d_H is the Hamming distance, and L is the fingerprint length). Sequences with a Jaccard index above a defined threshold are grouped into connected components for coarse clustering. Clusters with fewer than 500 sequences then undergo direct pairwise comparisons using normalized Levenshtein distance calculations (Levenshtein 1965). Larger clusters are processed using a k-mer frequency approach, where sequences are converted into normalized 4-mer count vectors, and Faiss's inner product search identifies candidate matches (Ren et al. 2018). Only sequence pairs passing k-mer similarity and length difference filters undergo final normalized Levenshtein distance verification.

1.5 Assignment

1.5.1 Multi-mapping fragment reassignment

Reassignment of multi-mapping fragments proceeds in three steps. First, intmap uses Locality-Sensitive MinHashing to build coarse clusters of multi-mapping fragments based on approximate $5'$ sequence similarity (Broder et al. 2000; Indyk and Motwani 1998). These coarse clusters are subsequently refined using normalized Levenshtein distance calculations to assess true sequence similarity. Sequence similarities are computed from the $5'$ ends of the two sequences being compared up to the length of the shorter of the two sequences. Next, intmap individually reassigns the fragments in each cluster based on sequence similarity with the sequences of uniquely-mapping fragments. Remaining multi-mapping fragments in each cluster are then conditionally reassigned to a single probable position *en masse* based on a weighted sampling of mapped positions within their respective cluster. Clusters that fail to meet the multi-mapping group size threshold (defined as a fraction of total deduplicated multi-mapping reads) are not reassigned, as they are unlikely to harbor any information on positionally-biased ISs.

It is important to note that this two-pronged approach to multi-mapping fragment re-assignment will not unequivocally assign multi-mapping fragments to the "correct" genomic position. It will, however, improve the accuracy of multi-mapping fragment position assignment when a multi-mapping fragment is derived from an otherwise uniquely-mapped position or when a positionally-biased population is located at a genomic locus characterized by repetitive or low-complexity sequences.

1.5.2 Final pass

To further reduce mapping artifacts, intmap performs a final pass through the assigned fragment positions to 1) identify low-abundance sites within a user-defined window of high-abundance sites and 2) filter out low-confidence sites. The count-based definition of a high-abundance site is user-defined and can be adapted to different analytical needs. If the abundance-level of a high-abundance site is above a user-defined fold-change threshold of a nearby low-abundance site, the low-abundance site

is reassigned to the high-abundance position. Similar position-based criteria for collapsing nearby ISs have been used in prior IS mapping software (Wells et al. 2020; Yan et al. 2023; Sherman et al. 2017). Similarly, intmap identifies low-confidence sites according to a fold-change threshold relative to the average number of times individual ISs were sequenced in an experiment. Sites sequenced less-than the fold-change threshold of the average value are deemed low-confidence and removed.

1.6 Auxilliary functions and helper scripts

intmap contains several functionalities that are not strictly required for IS mapping, but that can aid in data analysis and/or reducing experimental artifacts. These include: an implementation of the SonicAbundance method, previously described in Berry et al. (2017), to estimate IS abundance using maximum likelihood estimation (MLE); a method similar to the methods described in Wells et al. (2020) and Breton et al. (2020) to look for and remove fragments likely arising from mispriming during library preparation; functionality to look for patterned UMIs, as described in Yan et al. (2023), to reinforce UMI fidelity; functionality to derive and/or verify integrant- and linker-end consensus sequences from raw data; an option to quantify IS overlap with/proximity to user-defined genomic features; and an option to summarize AAV integration hot spots from the mapped ISs.

intmap additionally comes with two helper scripts that are automatically installed with the intmap conda environment. The first of these, intwrap, is a simple wrapper for running intmap_demux and intmap_multi together in a single command. The second is prepim, a helper script that aids in preparation of the setup file used by intmap_multi. Both helper scripts are callable directly from the command line. More information is provided in the GitHub repository.

1.7 Outputs

intmap creates a new "processed" directory within the working directory. Within "processed", intmap creates five subdirectories: "alignments", "cropped_reads", "fragments", "removed", and "sites". "Alignments" stores the BAM and BAI files for all analyses. "Cropped_reads" stores the FASTQ files generated after removal of integrant and linker sequences from input data. "Fragments" stores BED files for the final mapped fragments and summary files containing read name, fragment coordinates, fragment counts, fragment type (unique, multi-mapping, multi-mapping reassigned, etc.), identified integrant/linker sequences, UMI sequences, and IS coordinates. "Removed" stores JSON files denoting kept fragment read names and the read names of all associated fuzzy duplicates. "Removed" additionally stores summary files similar to the one in "fragments" for any/all fragments reassigned during the final pass. "Sites" stores BED files for all mapped ISs and all unique ISs (i.e., distinct ISs; this is unrelated to the uniquely-mapping fragments described above). The score column in the BED file for all mapped sites stores the number of duplicates observed for the fragment corresponding to that particular IS. The score column in the BED file for unique sites stores the number of distinct fragments mapped for each distinct site. When genomic annotation files are given to intmap, an additional "comparisons" subdirectory is made. This directory stores BED files denoting ISs overlapping each genomic feature-set and the distance from each mapped IS to the nearest genomic feature in each provided feature-set, respectively. Similarly, when the 'write_peaks' flag is set, intmap will create a "peaks" subdirectory, which stores for each dataset a BED file containing summarized peak coordinates, along with the coordinates of each peak apex. All files are named according to the name given to intmap. All coordinate-containing output files are in the BEDn+ format (https://github.com/samtools/hts-specs/blob/master/BEDv1.pdf). The structure

of the output directory tree is intended to facilitate navigable analysis of multiple datasets in the same directory.

2 Results

2.1 Benchmarking

2.1.1 Simulated short-read paired-end data with fixed termini

As an initial benchmark, we generated a dataset *in silico* containing 1 million genomic fragments from the human genome (genome build hs1; Nurk et al. (2022)) with a mean fragment length of 500 bp and Poisson variance. Target integrant and linker sequences were appended to 50% of these fragments. These comprise the ground truth population expected to be identified by intmap. Non-target integrant and linker sequences were appended to the remaining 50% of fragments. To simulate the presence of a clonal population, 25% of the 500,000 target fragments were derived from a single genomic position. Finally, 300 bp paired-end FASTQ files were generated from these fragments. Using these FASTQ files, we quantified intmap runtime and assessed mapping accuracy.

Runtime was initially quantified using intmap's most computationally involved mapping routine. To assess the performance improvement gained from parallelization, we performed identical analyses of the simulated data over an increasing numbers of cores on an Apple M4 MacBook Pro with 48 GB of RAM. Runtime was assessed for each major processing step, as well as overall. On a single core, the analysis completed in 31.3 minutes (Figure S1A). As the number of cores used in an analysis increased, runtime decreased up to 5.9-fold (5.3 minutes on 10 cores; Figure S1A). The alignment and multi-mapping fragment deduplication steps were the most time-intensive steps in the intmap workflow (Figure S1A).

Next, we compared intmap results for 1) uniquely mapping fragments only and 2) uniquely- and multi-mapping fragments together with multi-mapping fragment reassignment. The overall runtimes were 4.8 and 6.7 minutes, respectively (Figure S1B). For comparison, the runtime for mapping uniquely- and multi-mapping fragments together without multi-mapping fragment reassignment was 5.9 minutes. Thus, inclusion of multi-mapping fragments does carry a runtime penalty, but it is not dramatic. We then quantified the percentage of expected fragments that were retained after cropping, alignment, QC, and deduplication, as well as the percentage of mapped sites that were mapped to the correct genomic positions. Both analyses retained all 500,000 expected fragments after the cropping and alignment steps (Figure S1C). Moreover, both analysis conditions retained 478,162 uniquely-mapping fragments after QC (96.6% of expected; Figure S1C). Of these, 478,156 were retained after deduplication and 478,108 ($>$ 99.9% of deduplicated uniquely-mapping fragments) were mapped to the correct genomic positions (Figure S1C). Inclusion of multi-mapping fragments increased the number of fragments retained after QC to 499,844 ($>$ 99.9% expected) and the total number of deduplicated fragments to 499,838 ($>$ 99.9% expected; Figure S1C). Of these, 483,898 were mapped to the correct genomic positions (96.8% of deduplicated fragments; Figure S1C). The decrease in overall mapping accuracy upon inclusion of multi-mapping fragments is due to the positional degeneracy of these fragments. Both analysis-types correctly mapped the expected clonal population (Figure S1D). Importantly, the positional ambiguity of multi-mapping fragments does

not obfuscate all biological interpretability. The percent overlap of ISs derived from non-clonal multi-mapping fragments with various genomic annotations, such as genes, speckle-associated domains (SPADs; Chen et al. (2018), Zhang et al. (2021)), lamina-associated domains (LADs; Meuleman et al. (2013), Kind et al. (2015)), and centromere/satellite regions (CenSat; Altemose et al. (2022)) can inform the mapping accuracy of ISs within larger genomic regions. Despite having an overall mapping accuracy of just 26.7% at the nucleotide-level, the percent overlap of multi-mapping ISs with the aforementioned genomic annotations were nearly identical to ground-truth values (Figure S1E). These results highlight that even though the exact location of multi-mapping fragments can be difficult to define, valuable information can still be gleaned from these data.

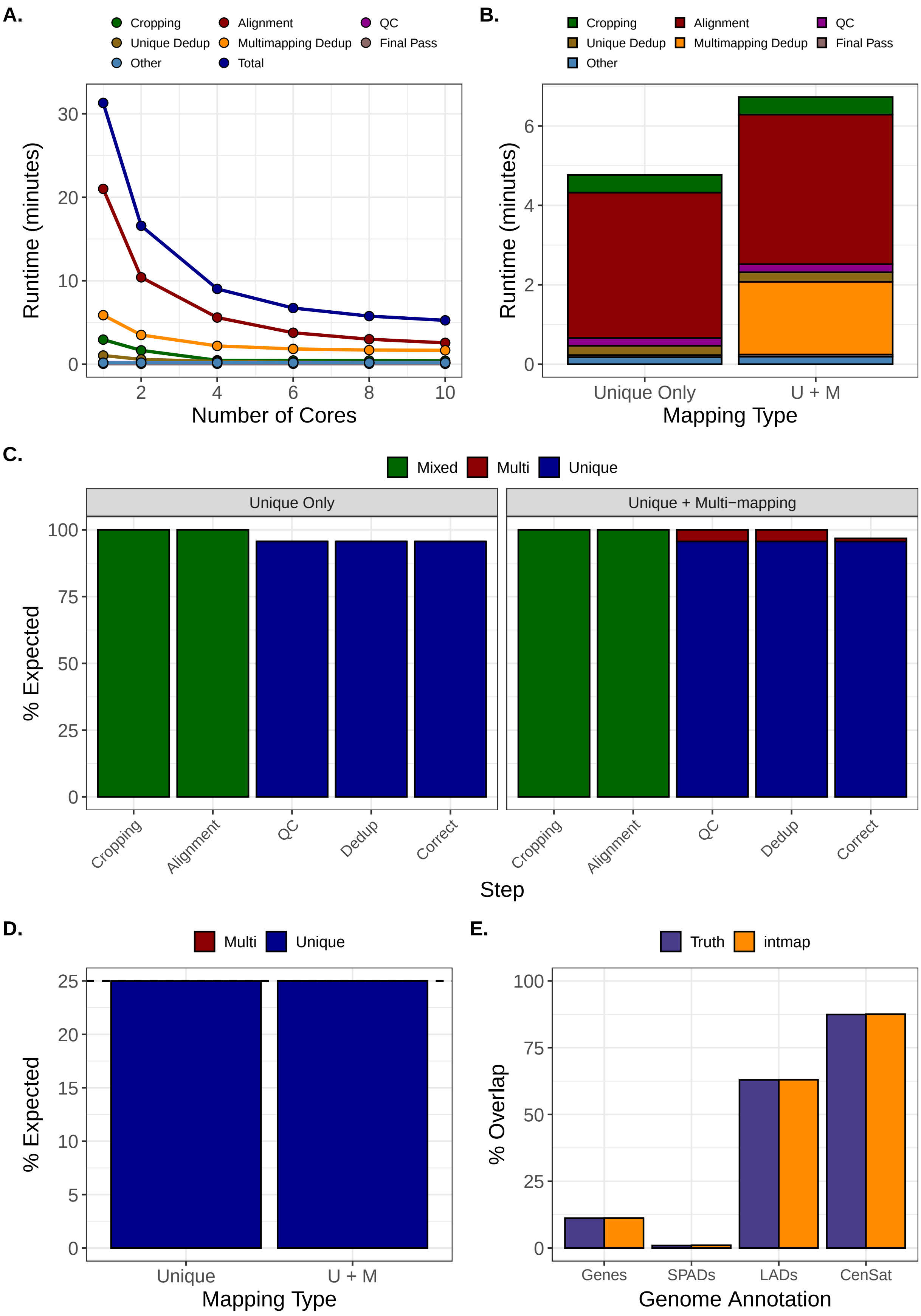
A.
Cropping
Alignment
QC
Unique Dedup
Multimapping Dedup
Final Pass
Other
Total
Runtime (minutes)
Number of Cores
B.
Runtime (minutes)
Unique Only
U + M
Mapping Type
C.
Mixed
Multi
Unique
Unique Only
Unique + Multi−mapping
% Expected
Cropping
Alignment
QC
Dedup
Correct
Step
D.
% Expected
Unique
U + M
Mapping Type
E.
Truth
intmap
% Overlap
Genes
SPADs
LADs
CenSat
Genome Annotation

Figure S1: Benchmarking with paired-end, short-read data. A. Total and per-step runtimes for short-read paired-end data on the indicated number of cores. B. Total and per-step runtimes for mapping with uniquely-mapping fragments only (Unique Only) and with both uniquely-mapping and multi-mapping fragments (U + M). All analyses were performed across 6 cores. C. The percentage of expected fragments (the 500,000 ground-truth fragments) retained after each of the indicated processing steps, along with the percentage of correctly mapped sites. The bars are colored according to the relative contributions of each fragment type. When unique and multi-mapping fragments are undifferentiated (i.e., before QC), they are referred to as “mixed”. D. Clonal abundance estimates from each mapping condition. The true abundance level of 25% is indicated by the dashed line. Bars are colored according to the relative contributions of uniquely-mapping and multi-mapping fragments. E. The percent overlap of non-clonal sites derived from multi-mapping fragments with the indicated genomic annotations. The mapped positions (orange) are compared to ground-truth positions (purple).

2.1.2 Other data-types with fixed termini

While paired-end data is the current standard for IS mapping, studies published as recently as 2022 have used single-end short-read sequencing data to map retroviral ISs from infected cells (Winans et al. 2022). In addition, the improvement and increasing availability of both the Oxford Nanopore and PacBio sequencing platforms has led to the introduction of long-read sequencing to studies of genomic integration (Ivančić et al. 2022; Yasir et al. 2022; Artesi et al. 2021; Sadri et al. 2026). We assessed runtime and mapping accuracy for both single-end short-read and long-read simulated data. For short-read input data, we appended integrant-end UMIs to the first mate of the simulated paired-end data described above. For long-read input data, we expanded the target fragments used to generate the paired-end data described above to a mean fragment length of 5000 nucleotides and generated a new FASTQ file containing the entirety of each fragment. The respective analyses for both data-types included the retention of multi-mapping fragments and multi-mapping fragment relocation. The total runtime for single-end short-read mapping was 7.5 minutes (Figure S2A). The single-end short-read data contained 7-fold more multi-mapping fragments than the matched paired-end data. This resulted in a 2.9-fold increase in the runtime for multi-mapping fragment deduplication (compare the orange segments in Figures S1B and S2A). This is the reason for the longer total runtime for the single-end short-read data relative to the paired-end data. The total runtime for long-read data was 14.9 minutes (Figure S2A). The alignment step in the long-read analysis accounted for 58.5%, of the total runtime, making it the primary driver of the observed runtime increase relative to short-read data (Figure S2A). The percentage of expected fragments retained after cropping, alignment, QC, and deduplication for single-end short-read and long-read data closely mirrored the results for paired-end data (Figure S2A). intmap retains nearly all of the expected fragments through deduplication ($\geq$ 99.9% of expected for both; Figure S2B). The overall mapping accuracies were 95.9% and 99.4% of deduplicated fragments for single-end short-read and long-read data, respectively (Figure S2B). Clonal abundance estimates were 24.9% for single-end short-read data and $>$ 24.9% for long-read data (Figure S2C). Notably, the clonal population identified in the single-end short-read data was composed entirely of multi-mapping fragments, while the clonal population identified in the long-read data was composed entirely of uniquely-mapping fragments (Figure S2C). This difference highlights intmap’s ability to accurately identify clonal populations from ambiguously mapped data and underscores the utility of intmap’s multi-mapping fragment grouping and relocation strategy.

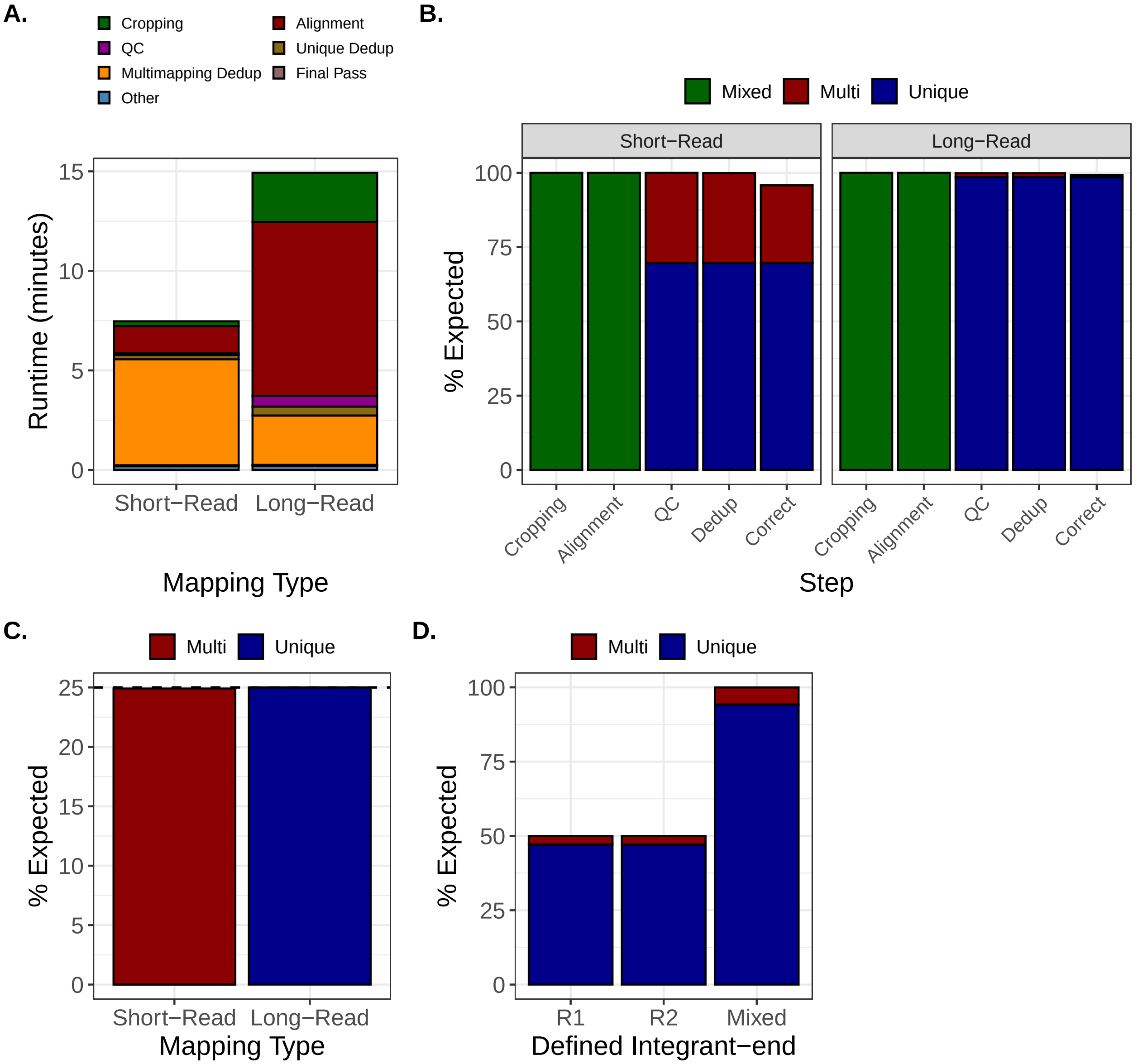

Figure S2: Analysis of other fixed-end data-types. A. Total and per-step runtimes for analysis of single-end short-read and long-read data using intmap. Both analyses were done over 6 cores. B. The percentage of expected fragments retained after each of the indicated processing steps, and the percentage of correctly mapped fragments, for both of the indicated data-types. The bars are colored according to relative contributions of each fragment-type. C. Clonal abundance estimates for both of the indicated data-types. The true abundance level is indicated by the dashed line. Bars are colored according to the relative contributions of uniquely-mapping and multi-mapping fragments. D. IS mapping from ISLA-type (directionally agnostic) data. intmap's 'mixed' mode was compared to analyses with reads 1 (R1) and 2 (R2) defined as the integrant-ends, respectively. Bars show the number of mapped ISs as a percentage of expected and are colored according to fragment-type.

For limited patient-derived samples from people living with HIV, integration site loop amplification (ISLA) is commonly used to map host-integrant junctions (Wagner et al. 2014; Einkauf et al. 2019, 2022; Jiang et al. 2020; Lian et al. 2023; Gasca-Capote et al. 2024; Vela et al. 2025). In this sequencing approach, random oligomers tagged with a portion of the integrant's own terminal-end

priming sequence are used to selectively linearly amplify the host-integrant junction (Wagner et al. 2014). Upon further amplification of the junction fragment using the designed integrant-end primer, a linear DNA fragment is synthesized harboring the integrant-end on one end and the reverse compliment of the terminal-end priming sequence on the other. This newly synthesized fragment supports loop formation that can then be extended (Wagner et al. 2014). Upon denaturation, the final product is a linear DNA molecule harboring the integrant-end on one terminus and the reverse compliment of the integrant starting with the terminal-end priming sequence on the other (Wagner et al. 2014). This product can subsequently be amplified using a single primer compatible with both ends of the molecule (Wagner et al. 2014). Unlike with L(A)M-PCR approaches for IS library preparation, there is no way to enforce directionality when appending sequencing adapters to ISLA products. This results in a mixed population wherein integrant-end sequences can be present on either read of a read pair or either end of a long-read fragment. One approach to handling this situation would be to analyze each dataset twice, swapping the read file designated as integrant-end reads between analyses. However, this is cumbersome and care must be taken to avoid the identification of false positives in the repeated searching for integrant- and linker-ends. intmap incorporates a 'mixed' flag that, when set, elegantly handles this situation. We demonstrate this with simulated data constructed to mimic the structure of ISLA-derived sequencing data (Wagner et al. 2014). In these data, 50% of the reads have integrant-ends on read 1 and 50% have integrant-reads on read 2. Moreover, the "linker" sequence on these data is the reverse compliment of the left-most 20 bp of the integrant-end fragment appended to integrant-end reads. When these data were analyzed without accounting for the mixed nature of the data, only ~50% of the reads were identified regardless of whether read 1 or read 2 was considered the integrant-end read (Figure S2D). When the 'mixed' flag was set, however, nearly all ($> 99.9\%$) of the reads were properly identified (Figure S2D), highlighting intmap's applicability to ISLA-type IS data.

2.1.3 Real-world data with fixed termini

We further benchmarked intmap against real-world IS data published alongside the INSPIIRED software (Sherman et al. 2017; Berry et al. 2017). We performed a head-to-head comparison of intmap with INSPIIRED's intSiteCaller module, which demultiplexes multiplexed IS data, removes integrant and linker sequences from the reads, aligns the reads to the host genome, and returns the positions of mapped IS coordinates (Sherman et al. 2017; Berry et al. 2017). Thus, intSiteCaller is the most direct analog of intmap in the INSPIIRED workflow. intSiteCaller maps and retains both uniquely- and multi-mapping fragments. As such, we similarly retained multi-mapping reads in our analysis. We additionally enabled multi-mapping fragment reassignment, as the authors of INSPIIRED noted that one of the expected clones is comprised of both uniquely- and multi-mapping fragments (Sherman et al. 2017). Importantly, intSiteCaller sequesters uniquely- and multi-mapping fragments into separate outputs, requiring manual reassignment, if desired. Moreover, while linker-end UMIs were present on INSPIIRED data, the INSPIIRED pipeline does not utilize UMIs for clonal abundance estimation (Sherman et al. 2017). INSPIIRED's authors instead prefer an MLE-based approach that they previously described (Sherman et al. 2017; Berry et al. 2017, 2012). As UMI information was available from INSPIIRED, however, we estimated IS abundances using both UMI- and MLE-based approaches. For INSPIIRED-derived sites, MLE-based abundance estimates were made using the hiReadsProcessor R package, which itself utilizes Berry's original sonicLength package (Malani 2025; Berry et al. 2012). intmap-derived MLE-based abundance estimates were made by re-running intmap with the 'mle' flag set. For UMI-based abundance estimates, INSPIIRED-derived sites were filtered to keep only a single representative of each UMI.

intmap's built-in UMI deduplication routine was used for UMI-based abundance estimates with intmap. The data used to compare intmap and INSPIIRED consisted of 5 distinct singularly clonal IS populations and 3 pooled polyclonal populations composed of known amounts of the 5 singularly clonal populations. Each population was additionally represented in quadruplicate, yielding a total of 32 distinct samples. The paired-end reads were structured such that the integrant-end was sequenced on Read 2 of a pair and the linker-end was sequenced on Read 1. Sample indexes for demultiplexing were moreover given in a separate index (I1) FASTQ file. We analyzed these data using a single core with 12 GB RAM. The total runtimes for these analyses — both demultiplexing and mapping — were 120.9 minutes and 18.4 minutes for INSPIIRED and intmap, respectively. Overall, intmap and INSPIIRED mapped similar numbers of sites in each sample, though some systematic differences were observed (Figure S3A). For all populations except C2, intmap mapped slightly more sites than INSPIIRED. This was due primarily to differences in the default analysis parameters in the respective software (e.g., exact integrant-end matching versus error-tolerant integrant-end matching). Both software support some degree of flexibility in this respect, but intmap in particular is highly adjustable. The clonal abundance estimates derived using both software were extremely similar (Figures S3B-C). This was true for both UMI- and MLE-based approaches (Figures S5B-C). Thus, both software are generally capable of accurately identifying the correct IS populations from infected cells. At the same time, we note that intmap was several-fold faster than INSPIIRED. It is also worth noting that both intmap and INSPIIRED mapped the clonal position in C2 (chr17:77440128:+) as a mixture of uniquely- and multi-mapping fragments. Both software additionally mapped at least one uniquely-mapping fragment in each C2 replicate to a spurious position at chr17_random:2406346:+ that is highly degenerate with the clonal position. Crucially, intmap's multi-mapping fragment relocation strategy correctly assigned the vast majority (95.9%) of mapped multi-mapping fragments to the expected clonal position instead of the degenerate competitor. This highlights the robustness of intmap's automated approach to assigning multi-mapping fragments to clonal positions.

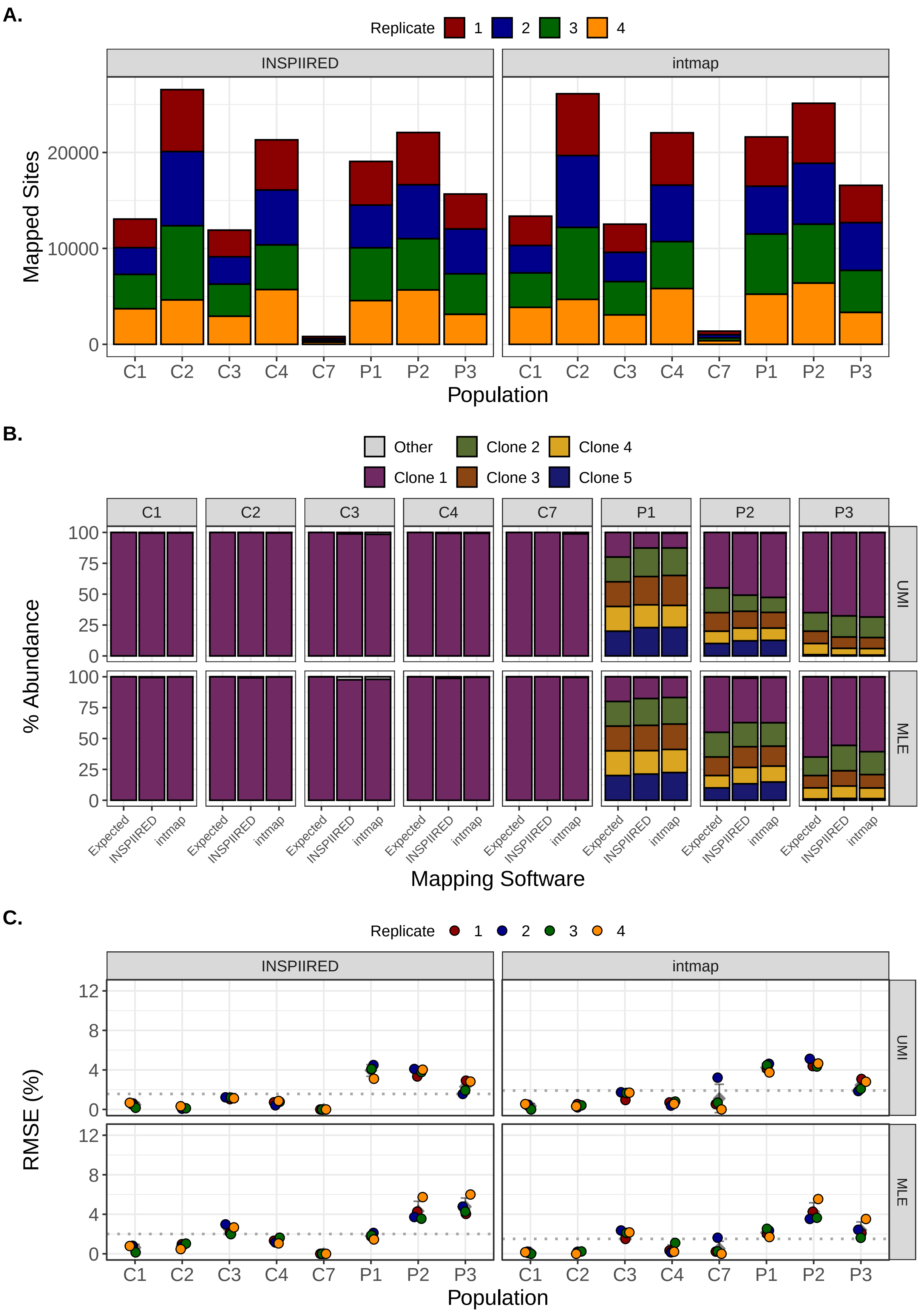
A.
Replicate 1 2 3 4
INSPIIRED
intmap
Mapped Sites
20000
10000
0
C1 C2 C3 C4 C7 P1 P2 P3
Population
B.
Other
Clone 1
Clone 2
Clone 3
Clone 4
Clone 5
C1 C2 C3 C4 C7 P1 P2 P3
% Abundance
100 75 50 25 0
UMI
MLE
Expected
INSPIIRED
intmap
Mapping Software
C.
Replicate 1 2 3 4
INSPIIRED
intmap
RMSE (%)
12 8 4 0
UMI
MLE
C1 C2 C3 C4 C7 P1 P2 P3
Population

Figure S3: Comparisons between intmap and INSPIIRED. A. The number of sites mapped by INSPIIRED and intmap. The respective populations are indicated on the x-axis. Each bar is the composite of four distinct replicates, with the number of sites belonging to each replicate being indicated by color. The total bar height is the total number of sites mapped for a given population across all replicates. B. Average clonal abundance estimates for each population (columns) according to expectation, INSPIIRED, and intmap. The two abundance estimation methods (UMI and MLE) are stratified by row. Bars are colored according to clone identity in each population. C. The root mean squared error (RMSE) of clonal abundance estimates for each population. Each replicate is plotted as a separate point. The average RMSE and associated standard deviation for each population are plotted as the gray diamonds and associated error bars. The overall average RMSE across each software and abundance estimation method is plotted in each facet as a horizontal dotted line.

To further demonstrate intmap's inherent flexibility, we analyzed a second real-world dataset published with the software IS-Seq (Yan et al. 2023). The library architecture expected by IS-Seq is different from the library architecture expected by INSPIIRED. The IS-Seq data contained sample-specific in-line barcodes on both the integrant- and linker-end reads (Yan et al. 2023). Moreover, the integrant-end was sequenced on Read 1 of a pair, while the linker-end was sequenced on Read 2. Despite these differences with the INSPIIRED data, intmap analyzed both datasets with ease. The IS-Seq data were comprised of sequences from 9 single-cell clones harboring between 1 and 5 unique lentiviral ISs each, as determined by vector copy number (VCN) estimation (Yan et al. 2023). Samples were demultiplexed with intmap_demux and serially analyzed with intmap_multi. To make the intmap analysis of IS-Seq data more "IS-Seq-like", we enforced a match between read UMIs and the expected UMI pattern encoded in the linker design (Yan et al. 2023). We additionally turned off retention of multi-mapping reads, as IS-Seq requires a MAPQ score ≥ 30 for reads that overlap repeat regions of the host genome (Yan et al. 2023). Lastly, given the expected clonality of these data and their relatively high sequencing depth, we defined low-confidence reads as any read sequenced less-than 10-fold the number of times the average IS was sequenced. The cumulative runtime for demultiplexing and analyzing these data was 34 minutes (3.8 minutes per sample, on average) utilizing 6 cores on an M4 Macbook Pro with 48 GB RAM. Figure S4A shows the clonal abundance estimates for each sample derived from IS-Seq and intmap, respectively, using both UMI- and MLE-based abundance estimates. Empirical abundance estimates are compared to the "expected" values derived from VCN estimation (e.g., VCN = 2 implies two clonal ISs with 50% abundance each). The associated RMSE values for each sample using both mapping software and abundance estimation techniques are shown in Figure S4B. The two software returned similar results overall (Figure S4A-B). For Sample E6, however, intmap identified noticably more "Other" sites than IS-Seq (Figure S4A). The E6 clonal fractions estimated by intmap and IS-Seq, respectively, were 55.0% and 59.5% for clone 1 and 37.7% and 39.6% for clone 2. Thus, the respective clonal popluations identified by the two software do not differ substantially. The simplest explanation for the observed difference, therefore, is that certain reads retained by intmap (using these analysis parameters) were filtered out by IS-Seq. Notably, the IS-Seq MLE-based E6 abundance estimates closely matched the intmap results. Regardless of the underlying reasons for these differences, their practical effects were small. The RMSE distributions for the IS-Seq and intmap analyses were very similar (Figure S4B). Both IS-Seq and intmap reliably estimated the expected IS distributions from these data.

Together, comparisons of intmap with both INSPIIRED and IS-Seq highlight that while all three software perform well at the stated task of mapping ISs from NGS data, intmap is the most flexible. The library designs of INSPIIRED and IS-Seq differ in key ways. Nevertheless, intmap accurately demultiplexed and mapped data generated for both "out-of-the-box". The tunability of intmap's

analysis parameters relative to both IS-Seq and INSPIIRED additionally make intmap compatible with a wide variety of IS data and highly adaptable to the nuances of individual datasets.

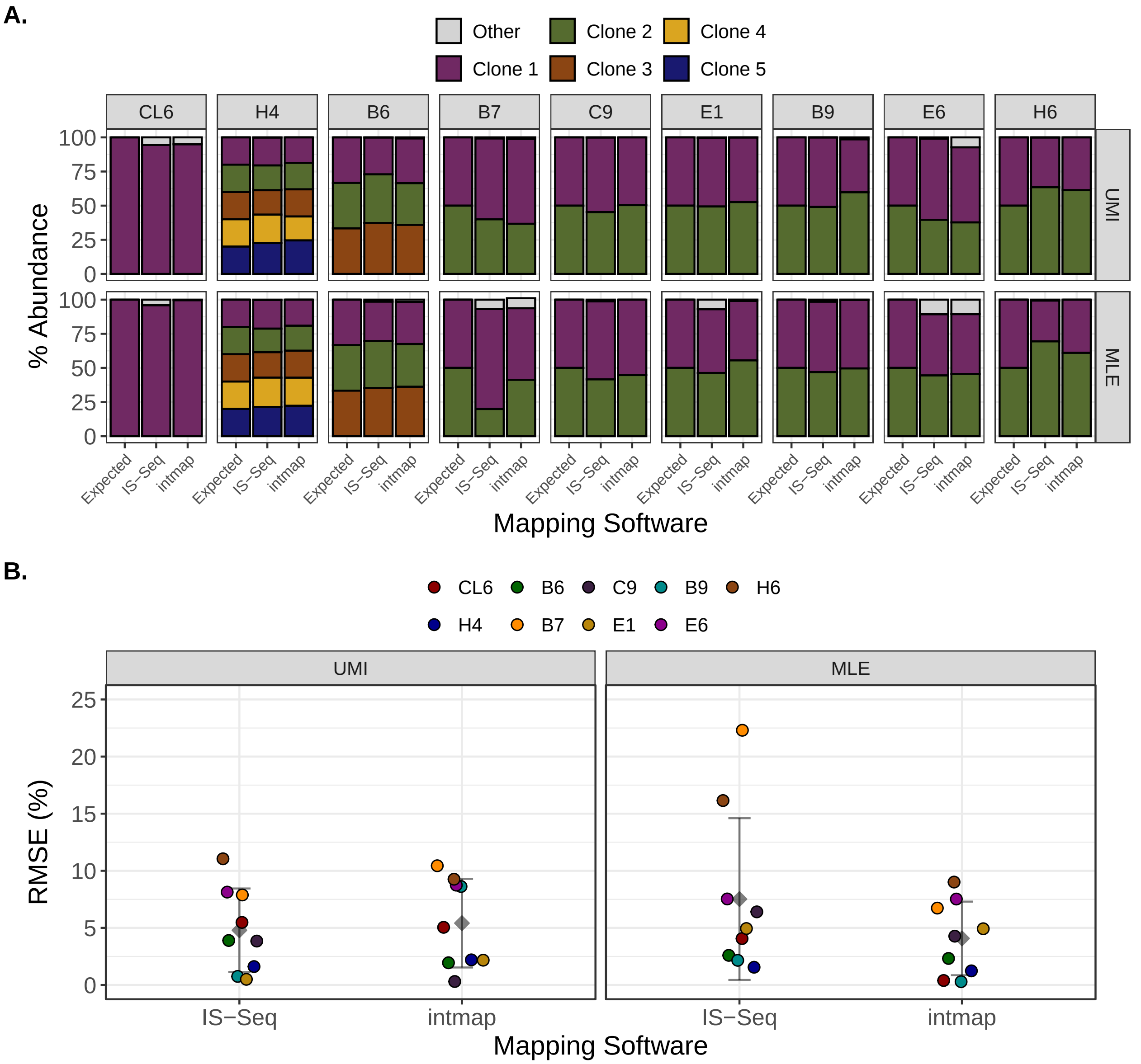

Figure S4: Comparisons between intmap and IS-Seq. A. Clonal abundance estimates for each sample (columns) according to expecatation, IS-Seq, and intmap, as indicated. UMI- and MLE-based abundance estimates from both mapping software are stratified by row. Bars are colored according to clone identity in each population. C. Root mean squared error (RMSE) of clonal abundance estimates for both mapping software. Per-sample RMSE values are plotted as colored points. The average RMSE and associated standard deviations for each software and abundance estimation procedure are plotted in gray.

2.1.4 Truncated integrant ends

Some integrating systems exhibit dramatic—often unpredictable—trunction of integrant ends during integration. One such system is AAV, for which data has shown truncations throughout the integrant's inverted terminal repeats (ITRs) (Nakai et al. 2005; Nguyen et al. 2021). In addition to truncated termini, AAV ITRs can exist in two possible sequence orientations, "flip" and "flop", which differ in the order of specific motifs that comprise the ITR (Wilmott et al. 2019). The lack of well-defined integrant ends make the identification of AAV integration events inherently more complicated than identification of, e.g., retroviral integrations. Given the (pseudo-)stochastic nature of integrant-end truncation, small regions of microhomology between the integrant-end and the host genome can blur integrant-host junctions. For example, consider a full-length integrant terminus that ends in the sequence 5′-ATGC-3′. If that sequence is truncated during integration to 5′-ATG-3′ and we assume equal nucleotide representation, there is a 25% chance that the host nucleotide at the integrant-host junction is also a C. In this instance, it would be impossible to unequivocally determine *post hoc* whether that C originated from the integrant or the host. It is useful to quantitatively define this inherent ambiguity. Applying the same assumptions as above, the probability of encountering microhomologies of length k can be expressed as a geometric distribution with $p = 1 - 0.25 = 0.75$, $k = 0, 1, 2, ...,$ and $P(k) = (1-p)^k p$. Using this simple model, one would expect to properly identify the correct integrant-host junction ~75% of the time. Extending this further, one would expect to be off by 1 bp ~18.8% of the time, 2 bp ~4.7% of the time, etc. Any errors allowed in ITR matching would exacerbate this positional ambiguity. This scenario is borne out in simulated data. We generated *in silico* 1 million genomic fragments from the human genome. Of these, 50% harbored AAV-derived integrant-ends. The other 50% were noise. The AAV-derived sequences were truncated AAV2 ITRs spanning between 10 and 145 nucleotides. Both the flip or flop orientations of the ITR were represented. We then mapped IS positions with intmap enforcing a 0% integrant-end error rate. Comparing the length differences between the ground-truth and matched ITR sequences, we obtained a difference distribution that closely matched the theoretical generalization described above (Figure S5A, blue/purple bars and black points). Modifying the intmap analysis to allow a 10% integrant-end error rate, the difference distribution between matched and expected ITRs substantially broadened (Figure S5A, red bars). The impact of the ambiguity associated with truncated integrant-end identification was evident when assessing the overall mapping accuracy of intmap's analysis of the simulated data (allowing no errors in integrant-end matching). While the presence of truncated integrant ends had little-to-no bearing on intmap's ability to crop, align, process, and deduplicate IS data, the overall mapping accuracy was just 73.5% (76.9% for uniquely-mapping fragments only; Figure S5B). This was a marked departure from the 96.8% error rate observed with fixed-end integrants (Figure S1C). Relative to the expected positions, however, 99.96% of mapped ISs derived from uniquely-mapping fragments were within 5 bp of the ground-truth positions. This is consistent with the theoretical value of 99.98%. Thus, mapped ISs from integrants with truncated ends should be considered in the slightly "fuzzier" context in which they can be mapped. The intmap runtime for the simulated AAV dataset was 7 minutes, indicating that the more complex cropping routine for truncated integrant ends does not markedly increase runtime relative to fixed-end analyses.

We next extended our analyses of AAV integration to real-world data. To this end, we analyzed two datasets described in Breton et al. (2020). These data were derived from mice that were co-administered with two different AAV vectors harboring a *S. aureus* Cas9 gene and the gene for a single guide RNA targeting the Ass1 genomic locus, respectively (Breton et al. 2020). We note that these data were partially processed. Linker and UMI information, minimally, were missing from the

deposited reads. Nevertheless, the Read 2 reads of each read pair contained AAV ITR sequences originating from the defined ITR primer binding site. We required a minimum 30 bp match with the input ITR sequence. We show the distribution of identified ITR breakpoints from two separate mice in Figure S5C. ITR positions were indexed from the start of the stated primer binding site (Breton et al. 2020). In both mice, we observed substantial internal cleavage with a dominant peak 66 nucleotides from the start of the ITR (Figure S5C). This is consistent with a well-known recombination hotspot within the ITR sequence at the start of the B/B' hairpin (Janovitz, Sadelain, and Falck-Pedersen 2014; Breton et al. 2020). Thus, the ITR sequences identified by intmap showed good accordance with the published literature. Finally, using a peak-calling strategy generally similar to the one described in Breton et al. (2020), we mapped > 99.9% of kept sites in both mice to the targeted Ass1 locus. Moreover, the peak apex at the Ass1 locus in both animals was at the expected Cas9 cut site (chr2:31518656). Altogether, these analyses demonstrate intmap's ability to accurately map IS positions from integrants with truncated terminal repeats.

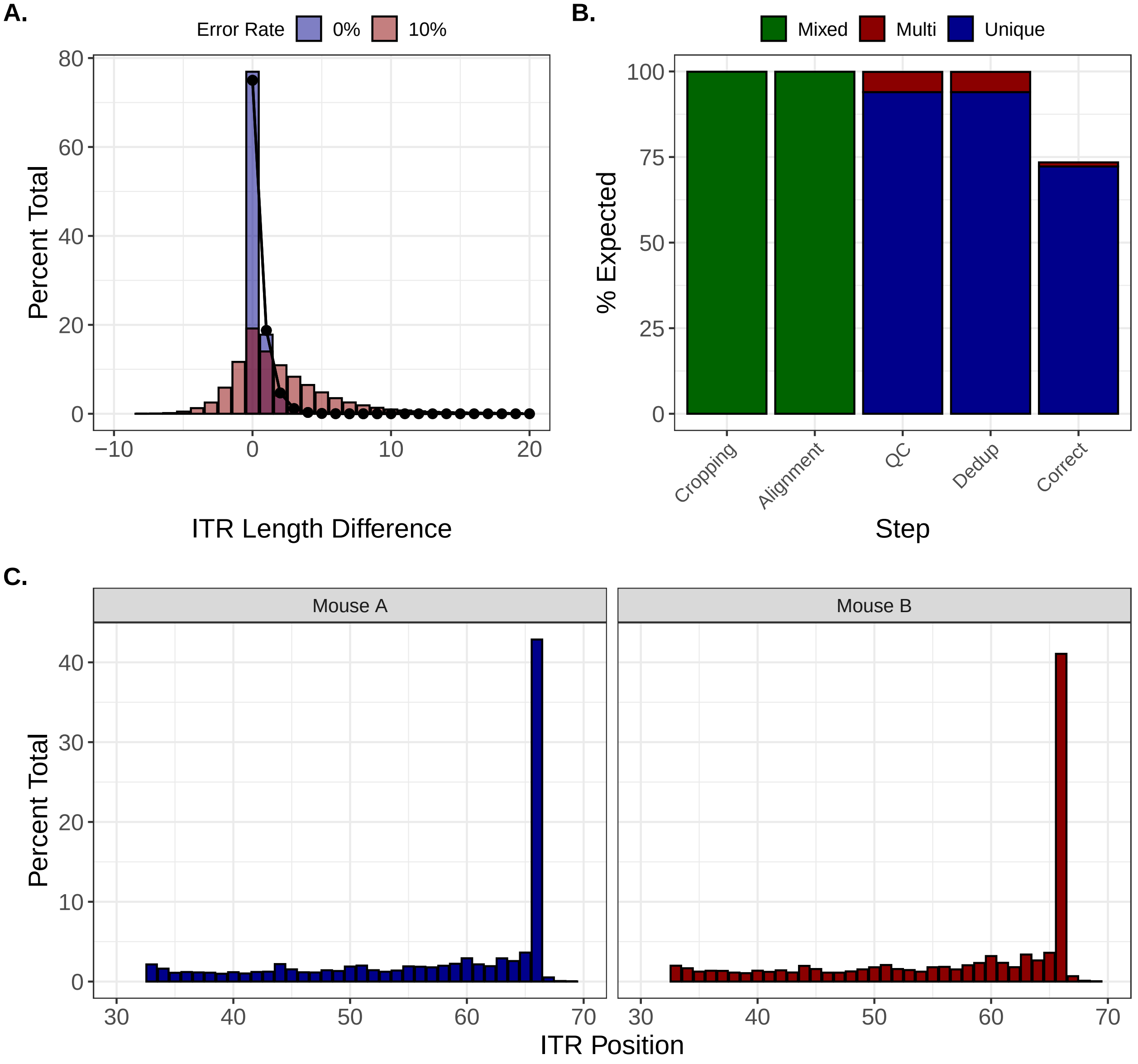

Figure S5: Truncated terminal repeats. A. ITR length comparisons between matched and expected lengths derived from simulated data. Data were analyzed allowing 0% (blue) and 10% (red) error rates in ITR matching, respectively. Overlapping portions of each histogram interact to appear purple in appearance. Theoretically expected proportions described in section 2.1.4 of the Supplement are shown as black points. B. Mapping summary and accuracy for the analysis of simulated data allowing a 0% ITR-end error rate. Bars are colored according to the relative contributions of each fragment type. C. Mapped ITR breakpoint positions from real-world data. Data from two separate mice are shown in each facet.

3 Supplemental Methods

3.1 Genomic annotations

Gene (JHU RefSeqv110 + Liftoff v5.2) and CenSat annotations for the hs1/CHM13v2 genome were downloaded from https://github.com/marbl/CHM13. The scripts used to define SPAD and LAD annotations in the hs1 genome can be found at https://github.com/gbedwell/chromatin_domains. These scripts follow the methods described in Zhang, *et. al.* and Meuleman, *et. al.* for SPAD and LAD calling, respectively (Zhang et al. 2021; Meuleman et al. 2013).

3.2 Integration site datasets

3.2.1 Simulated data

Simulated IS datasets were generated using xInt (https://github.com/gbedwell/xInt; currently under development). Custom bash scripts were used to concatenate the smaller generated datasets and to shuffle the concatenated output. The final shuffled FASTQ files were used as input data for the presented analyses. The ground-truth genomic coordinates for the simulated fragments were concatenated and stratified by type using a custom R script. Custom R scripts were additionally used to append UMIs to the single-end short-read simulated data and to expand the ground-truth genomic coordinates and fragments for long-read data simulation. All scripts, simulated data, and analysis outputs are provided on Zenodo.

3.2.2 Real data

Experimental data used for benchmarking was downloaded from the Sequence Read Archive (SRR3883183, SRR11212470, and SRR11212471) and the Gene Expression Omnibus (GSM6164817). The respective barcode sequences used for demultiplexing IS-Seq-associated data were obtained from Yan et al. (2023). Information pertaining to INSPIIRED-assocated data was found in the INSPIIRED GitHub repository (https://github.com/BushmanLab/INSPIIRED) and Sherman et al. (2017). All barcode files, analysis commands/parameters, and analysis outputs described here are included on Zenodo.

References

Altemose, Nicolas, Glennis A. Logsdon, Andrey V. Bzikadze, Pragya Sidhwani, Sasha A. Langley, Gina V. Caldas, Savannah J. Hoyt, et al. 2022. "Complete Genomic and Epigenetic Maps of Human Centromeres." *Science* 376 (6588): eabl4178. https://doi.org/10.1126/science.abl4178.

Artesi, Maria, Vincent Hahaut, Basiel Cole, Laurens Lambrechts, Fereshteh Ashrafi, Ambroise Marçais, Olivier Hermine, et al. 2021. "PCIP-seq: Simultaneous Sequencing of Integrated Viral Genomes and Their Insertion Sites with Long Reads." *Genome Biology* 22 (1): 97. https://doi.org/10.1186/s13059-021-02307-0.

Bachmann, Max. 2024. "RapidFuzz."

Barnett, Matthew. 2024. "Regex."

Berry, Charles C., Nicolas A. Gillet, Anat Melamed, Niall Gormley, Charles R. M. Bangham, and Frederic D. Bushman. 2012. "Estimating Abundances of Retroviral Insertion Sites from DNA Fragment Length Data." *Bioinformatics* 28 (6): 755–62. https://doi.org/10.1093/bioinformatics/bts004.

Berry, Charles C., Christopher Nobles, Emmanuelle Six, Yinghua Wu, Nirav Malani, Eric Sherman, Anatoly Dryga, et al. 2017. "INSPIIRED: Quantification and Visualization Tools for Analyzing Integration Site Distributions." *Molecular Therapy - Methods & Clinical Development* 4 (March): 17–26. https://doi.org/10.1016/j.omtm.2016.11.003.

Breton, Camilo, Peter M. Clark, Lili Wang, Jenny A. Greig, and James M. Wilson. 2020. "ITR-Seq, a Next-Generation Sequencing Assay, Identifies Genome-Wide DNA Editing Sites in Vivo Following Adeno-Associated Viral Vector-Mediated Genome Editing." *BMC Genomics* 21 (1): 239. https://doi.org/10.1186/s12864-020-6655-4.

Broder, Andrei Z, Moses Charikar, Alan M Frieze, and Michael Mitzenmacher. 2000. "Min-Wise Independent Permutations." *Journal of Computer and System Sciences* 60 (3): 630–59. https://doi.org/10.1006/jcss.1999.1690.

Chen, Yu, Yang Zhang, Yuchuan Wang, Liguo Zhang, Eva K. Brinkman, Stephen A. Adam, Robert Goldman, Bas van Steensel, Jian Ma, and Andrew S. Belmont. 2018. "Mapping 3D Genome Organization Relative to Nuclear Compartments Using TSA-Seq as a Cytological Ruler." *Journal of Cell Biology* 217 (11): 4025–48. https://doi.org/10.1083/jcb.201807108.

Cock, Peter J. A., Tiago Antao, Jeffrey T. Chang, Brad A. Chapman, Cymon J. Cox, Andrew Dalke, Iddo Friedberg, et al. 2009. "Biopython: Freely Available Python Tools for Computational Molecular Biology and Bioinformatics." *Bioinformatics* 25 (11): 1422–23. https://doi.org/10.1093/bioinformatics/btp163.

Cormen, Thomas H., ed. 2009. *Introduction to Algorithms*. 3. ed. Cambridge, Mass.: MIT Press.

Daily, Jeff. 2016. "Parasail: SIMD C Library for Global, Semi-Global, and Local Pairwise Sequence Alignments." *BMC Bioinformatics* 17 (1): 81. https://doi.org/10.1186/s12859-016-0930-z.

Danecek, Petr, James K Bonfield, Jennifer Liddle, John Marshall, Valeriu Ohan, Martin O Pollard, Andrew Whitwham, et al. 2021. "Twelve Years of SAMtools and BCFtools." *GigaScience* 10 (2): giab008. https://doi.org/10.1093/gigascience/giab008.

Douze, Matthijs, Alexandr Guzhva, Chengqi Deng, Jeff Johnson, Gergely Szilvasy, Pierre-Emmanuel Mazaré, Maria Lomeli, Lucas Hosseini, and Hervé Jégou. 2024. "The Faiss Library." arXiv. https://arxiv.org/abs/2401.08281.

Einkauf, Kevin B., Guinevere Q. Lee, Ce Gao, Radwa Sharaf, Xiaoming Sun, Stephane Hua, Samantha M. Y. Chen, et al. 2019. "Intact HIV-1 Proviruses Accumulate at Distinct Chromosomal Positions During Prolonged Antiretroviral Therapy." *Journal of Clinical Investigation* 129 (3): 988–98. https://doi.org/10.1172/JCI124291.

Einkauf, Kevin B., Matthew R. Osborn, Ce Gao, Weiwei Sun, Xiaoming Sun, Xiaodong Lian, Elizabeth M. Parsons, et al. 2022. "Parallel Analysis of Transcription, Integration, and Sequence of Single HIV-1 Proviruses." *Cell*, January, S0092867421014495. https://doi.org/10.1016/j.cell.2021.12.011.

Fox, Joseph. 2024. "Pybloom-Live."

Gasca-Capote, Carmen, Xiaodong Lian, Ce Gao, Isabelle C. Roseto, María Reyes Jiménez-León, Gregory Gladkov, María Inés Camacho-Sojo, et al. 2024. "The HIV-1 Reservoir Landscape in Persistent Elite Controllers and Transient Elite Controllers." *Journal of Clinical Investigation* 134 (8): e174215. https://doi.org/10.1172/JCI174215.

Hamming, R. W. 1950. "Error Detecting and Error Correcting Codes." *Bell System Technical Journal* 29 (2): 147–60. https://doi.org/10.1002/j.1538-7305.1950.tb00463.x.

Harris, Charles R., K. Jarrod Millman, Stéfan J. van der Walt, Ralf Gommers, Pauli Virtanen, David Cournapeau, Eric Wieser, et al. 2020. "Array Programming with NumPy." *Nature* 585 (7825): 357–62. https://doi.org/10.1038/s41586-020-2649-2.

Heng Li. 2025. "Seqtk: Toolkit for Processing Sequence in FASTA/Q Formats."

Indyk, Piotr, and Rajeev Motwani. 1998. "Approximate Nearest Neighbors: Towards Removing the Curse of Dimensionality." In *Proceedings of the Thirtieth Annual ACM Symposium on Theory of Computing - STOC '98*, 604–13. Dallas, Texas, United States: ACM Press. https://doi.org/10.1145/276698.276876.

Ivančić, Dimitrije, Júlia Mir-Pedrol, Jessica Jaraba-Wallace, Núria Rafel, Avencia Sanchez-Mejias, and Marc Güell. 2022. "INSERT-seq Enables High-Resolution Mapping of Genomically Integrated DNA Using Nanopore Sequencing." *Genome Biology* 23 (1). https://doi.org/10.1186/s13059-022-02778-9.

Janovitz, Tyler, Michel Sadelain, and Erik Falck-Pedersen. 2014. "Adeno-Associated Virus Type 2 Preferentially Integrates Single Genome Copies with Defined Breakpoints." *Virology Journal* 11 (1): 15. https://doi.org/10.1186/1743-422X-11-15.

Jiang, Chenyang, Xiaodong Lian, Ce Gao, Xiaoming Sun, Kevin B. Einkauf, Joshua M. Chevalier, Samantha M.Y. Chen, et al. 2020. "Distinct Viral Reservoirs in Individuals with Spontaneous Control of HIV-1." *Nature* 585 (7824): 261–67. https://doi.org/10.1038/s41586-020-2651-8.

Johnson, Jeff, Matthijs Douze, and Hervé Jégou. 2017. "Billion-Scale Similarity Search with GPUs." arXiv. https://doi.org/10.48550/arXiv.1702.08734.

Kind, Jop, Ludo Pagie, Sandra S. de Vries, Leila Nahidiazar, Siddharth S. Dey, Magda Bienko, Ye Zhan, et al. 2015. "Genome-Wide Maps of Nuclear Lamina Interactions in Single Human Cells." *Cell* 163 (1): 134–47. https://doi.org/10.1016/j.cell.2015.08.040.

Langmead, Ben, and Steven L Salzberg. 2012. "Fast Gapped-Read Alignment with Bowtie 2." *Nature Methods* 9 (4): 357–59. https://doi.org/10.1038/nmeth.1923.

Levenshtein, Vladimir I. 1965. "Binary Codes Capable of Correcting Deletions, Insertions, and Reversals." *Soviet Physics. Doklady* 10: 707–10.

Li, Heng. 2018. "Minimap2: Pairwise Alignment for Nucleotide Sequences." *Bioinformatics* 34 (18): 3094–3100. https://doi.org/10.1093/bioinformatics/bty191.

Li, Heng, Bob Handsaker, Alec Wysoker, Tim Fennell, Jue Ruan, Nils Homer, Gabor Marth, Goncalo Abecasis, Richard Durbin, and 1000 Genome Project Data Processing Subgroup. 2009. "The Sequence Alignment/Map Format and SAMtools." *Bioinformatics* 25 (16): 2078–79. https://doi.org/10.1093/bioinformatics/btp352.

Lian, Xiaodong, Kyra W. Seiger, Elizabeth M. Parsons, Ce Gao, Weiwei Sun, Gregory T. Gladkov, Isabelle C. Roseto, et al. 2023. "Progressive Transformation of the HIV-1 Reservoir Cell Profile over Two Decades of Antiviral Therapy." *Cell Host & Microbe* 31 (1): 83–96.e5. https://doi.org/10.1016/j.chom.2022.12.002.

Malani, Nirav V. 2025. *hiReadsProcessor: Functions to Process LM-PCR Reads from 454/Illumina Data*. Manual. https://doi.org/10.18129/B9.bioc.hiReadsProcessor.

Mariotti, Marco. n.d. "Pyranges V1: A Python Framework for Ultrafast Sequence Interval Operations."

Martin, Marcel. 2011. "Cutadapt Removes Adapter Sequences from High-Throughput Sequencing Reads." *EMBnet.journal* 17 (1): 10–12. https://doi.org/10.14806/ej.17.1.200.

McKinney, Wes. 2010. "Data Structures for Statistical Computing in Python." In *Proceedings of the 9th Python in Science Conference*, edited by Stéfan van der Walt and Jarrod Millman, 56–61. https://doi.org/10.25080/Majora-92bf1922-00a.

Meuleman, Wouter, Daan Peric-Hupkes, Jop Kind, Jean-Bernard Beaudry, Ludo Pagie, Manolis Kellis, Marcel Reinders, Lodewyk Wessels, and Bas Van Steensel. 2013. "Constitutive Nuclear Lamina–Genome Interactions Are Highly Conserved and Associated with A/T-rich Sequence." *Genome Research* 23 (2): 270–80. https://doi.org/10.1101/gr.141028.112.

Nakai, Hiroyuki, Xiaolin Wu, Sally Fuess, Theresa A. Storm, David Munroe, Eugenio Montini, Shawn M. Burgess, Markus Grompe, and Mark A. Kay. 2005. "Large-Scale Molecular Characterization of Adeno-Associated Virus Vector Integration in Mouse Liver." *Journal of Virology* 79 (6): 3606–14. https://doi.org/10.1128/JVI.79.6.3606-3614.2005.

Nguyen, Giang N., John K. Everett, Samita Kafle, Aoife M. Roche, Hayley E. Raymond, Jacob Leiby, Christian Wood, et al. 2021. "A Long-Term Study of AAV Gene Therapy in Dogs with Hemophilia A Identifies Clonal Expansions of Transduced Liver Cells." *Nature Biotechnology* 39 (1): 47–55. https://doi.org/10.1038/s41587-020-0741-7.

Nurk, Sergey, Sergey Koren, Arang Rhie, Mikko Rautiainen, Andrey V. Bzikadze, Alla Mikheenko, Mitchell R. Vollger, et al. 2022. "The Complete Sequence of a Human Genome." *Science (New York, N.Y.)* 376 (6588): 44–53. https://doi.org/10.1126/science.abj6987.

Quinlan, Aaron R., and Ira M. Hall. 2010. "BEDTools: A Flexible Suite of Utilities for Comparing Genomic Features." *Bioinformatics* 26 (6): 841–42. https://doi.org/10.1093/bioinformatics/btq033.

Ren, Jie, Xin Bai, Yang Young Lu, Kujin Tang, Ying Wang, Gesine Reinert, and Fengzhu Sun. 2018. "Alignment-Free Sequence Analysis and Applications." *Annual Review of Biomedical Data Science* 1 (July): 93–114. https://doi.org/10.1146/annurev-biodatasci-080917-013431.

Sadri, Ghazal, Steven T. Nadakal, William Lauer, Justin Kos, Parmit K. Singh, Erin Elliott, Catherine W. Kaiser, et al. 2026. "Development and Validation of HIV SMRTcap for the Characterization of HIV-1 Reservoirs Across Tissues and Subtypes." Edited by Jason M. Brenchley. *PLOS Pathogens* 22 (1): e1013171. https://doi.org/10.1371/journal.ppat.1013171.

Seabold, Skipper, and Josef Perktold. 2010. "Statsmodels: Econometric and Statistical Modeling with Python." In *Proceedings of the 9th Python in Science Conference*, edited by Stéfan van der Walt and Jarrod Millman, 92–96. https://doi.org/10.25080/Majora-92bf1922-011.

Senuma, Hajime. 2025. "Mmh3: A Python Extension for MurmurHash3." *Journal of Open Source Software* 10 (105): 6124. https://doi.org/10.21105/joss.06124.

Sherman, Eric, Christopher Nobles, Charles C. Berry, Emmanuelle Six, Yinghua Wu, Anatoly Dryga, Nirav Malani, et al. 2017. "INSPIIRED: A Pipeline for Quantitative Analysis of Sites of New DNA Integration in Cellular Genomes." *Molecular Therapy - Methods & Clinical Development* 4 (March): 39–49. https://doi.org/10.1016/j.omtm.2016.11.002.

Smith, Tom, Andreas Heger, and Ian Sudbery. 2017. "UMI-tools: Modeling Sequencing Errors in Unique Molecular Identifiers to Improve Quantification Accuracy." *Genome Research* 27 (3): 491–99. https://doi.org/10.1101/gr.209601.116.

The joblib Developers. 2024. "Joblib: Running Python Functions as Pipeline Jobs." Zenodo. https://doi.org/10.5281/zenodo.14915601.

The Pandas Development Team. 2024. "Pandas-Dev/Pandas: Pandas." Zenodo. https://doi.org/10.5281/zenodo.3509134.

The pysam Development Team. 2024. "Pysam."

Truong, Charles, Laurent Oudre, and Nicolas Vayatis. 2020. "Selective Review of Offline Change Point Detection Methods." *Signal Processing* 167 (February): 107299. https://doi.org/10.1016/j.sigpro.2019.107299.

Vela, Liliana C., Leah Carrere, Chloe Naasz, Sruthi Kalavacherla, Toong Seng Tan, Lesley De Armas, Ce Gao, et al. 2025. "Profound Reduction of HIV-1 Reservoir Cells over 3 Decades of Antiretroviral Therapy Started in Early Infancy." *JCI Insight* 10 (1): e186550. https://doi.org/10.1172/jci.insight.186550.

Virtanen, Pauli, Ralf Gommers, Travis E. Oliphant, Matt Haberland, Tyler Reddy, David Cournapeau, Evgeni Burovski, et al. 2020. "SciPy 1.0: Fundamental Algorithms for Scientific Computing in Python." *Nature Methods* 17: 261–72. https://doi.org/10.1038/s41592-019-0686-2.

Wagner, T. A., S. McLaughlin, K. Garg, C. Y. K. Cheung, B. B. Larsen, S. Styrchak, H. C. Huang, P. T. Edlefsen, J. I. Mullins, and L. M. Frenkel. 2014. "Proliferation of Cells with HIV Integrated into Cancer Genes Contributes to Persistent Infection." *Science* 345 (6196): 570–73. https://doi.org/10.1126/science.1256304.

Wells, Daria W., Shuang Guo, Wei Shao, Michael J. Bale, John M. Coffin, Stephen H. Hughes, and Xiaolin Wu. 2020. "An Analytical Pipeline for Identifying and Mapping the Integration Sites of HIV and Other Retroviruses." *BMC Genomics* 21 (1): 216. https://doi.org/10.1186/s12864-020-6647-4.

Wilmott, Patrick, Leszek Lisowski, Ian E. Alexander, and Grant J. Logan. 2019. "A User's Guide to the Inverted Terminal Repeats of Adeno-Associated Virus." *Human Gene Therapy Methods* 30 (6): 206–13. https://doi.org/10.1089/hgtb.2019.276.

Winans, Shelby, Hyun Jae Yu, Kenia De Los Santos, Gary Z. Wang, Vineet N. KewalRamani, and Stephen P. Goff. 2022. "A Point Mutation in HIV-1 Integrase Redirects Proviral Integration into Centromeric Repeats." *Nature Communications* 13 (1): 1474. https://doi.org/10.1038/s41467-022-29097-8.

Yamaguchi, Kota. 2024. "Faiss-Cpu."

Yan, Aimin, Cristina Baricordi, Quoc Nguyen, Luigi Barbarossa, Mariana Loperfido, and Luca Biasco. 2023. "IS-Seq: A Bioinformatics Pipeline for Integration Sites Analysis with Comprehensive Abundance Quantification Methods." *BMC Bioinformatics* 24 (1): 286. https://doi.org/10.1186/s12859-023-05390-1.

Yasir, Muhammad, A. Keith Turner, Martin Lott, Steven Rudder, David Baker, Sarah Bastkowski, Andrew J. Page, Mark A. Webber, and Ian G. Charles. 2022. "Long-Read Sequencing for Identification of Insertion Sites in Large Transposon Mutant Libraries." *Scientific Reports* 12 (1). https://doi.org/10.1038/s41598-022-07557-x.

Zhang, Liguo, Yang Zhang, Yu Chen, Omid Gholamalamdari, Yuchuan Wang, Jian Ma, and Andrew S. Belmont. 2021. "TSA-seq Reveals a Largely Conserved Genome Organization Relative to Nuclear Speckles with Small Position Changes Tightly Correlated with Gene Expression Changes." *Genome Research* 31 (2): 251–64. https://doi.org/10.1101/gr.266239.120.

Zhu, Eric, Vadim Markovtsev, Aleksey Astafiev, Arham Khan, Chris Ha, Wojciech Łukasiewicz, Adam Foster, et al. 2024. "Ekzhu/Datasketch: V1.6.5." Zenodo. https://doi.org/10.5281/zenodo.11462182.
